# Octanoate in Human Albumin Preparations Is Detrimental to Mesenchymal Stromal Cell Culture

**DOI:** 10.1155/2015/192576

**Published:** 2015-05-13

**Authors:** Way-Wua Wong, Andrew D. MacKenzie, Vicky J. Nelson, James M. Faed, Paul R. Turner

**Affiliations:** ^1^Spinal Cord Society New Zealand, Centre for Innovation, 87 St David Street, Dunedin 9054, New Zealand; ^2^Callaghan Innovation, 69 Gracefield Road, Lower Hutt 5040, New Zealand

## Abstract

Cell therapies hold great promise as the next major advance in medical treatment. To enable safe, effective *ex vivo* culture whilst maintaining cell phenotype, growth media constituents must be carefully controlled. We have used a chemically defined mesenchymal stromal cell culture medium to investigate the influence of different preparations of human serum albumin. We examined two aspects of cell culture, growth rate as measured by population doubling time and colony forming ability which is a representative measure of the stemness of the cell population. Albumin preparations showed comparative differences in both of these criteria. Analysis of the albumin bound fatty acids also showed differences depending on the manufacturing procedure used. We demonstrated that octanoate, an additive used to stabilize albumin during pasteurization, slows growth and lowers colony forming ability during *ex vivo* culture. Further to this we also found the level of Na^+^/K^+^ ATPase, a membrane bound cation pump inhibited by octanoate, is increased in cells exposed to this compound. We conclude that the inclusion of human serum albumin in *ex vivo* growth media requires careful consideration of not only the source of albumin, but also the associated molecular cargo, for optimal cell growth and behavior.

## 1. Introduction

Human bone marrow derived mesenchymal stromal cells (MSCs) have shown significant promise in clinical trials for the treatment of a variety of diseases [1]. They can be readily isolated and cultured* ex vivo* and trials have investigated the use of both autologous and allogeneic sources of these cells. To meet regulatory guidelines in many countries MSCs must be grown in media lacking high risk components such as bovine serum and this has led to the development of a selection of xenogeneic serum-free media cocktails. An important component of these media is some form of human serum albumin (hSA). Although commercially available media have confidential recipes it is highly likely they also contain some hSA. Albumin is a major protein in human plasma with a reference range of approximately 35–50 g/L and transports lipids, free fatty acids, bilirubin, metals, and some hormones around the body [2].

Albumin is obtained from human blood in two main ways: the Cohn fractionation process which exploits the differential solubility of plasma proteins in cold ethanol solutions in which ionic strength and pH are varied, or by column chromatographic methods (reviewed in [3]). Both of these processes are followed by a pasteurization step where albumin is stabilized by the addition of octanoate and heated to 60°C for 10 hours.

The main clinical use of albumin is as a plasma volume expander and the requirements for this function are quite different to those necessary in mesenchymal stromal cell culture. With this in mind we selected a defined cell culture medium, PPRF-msc6 [4], and measured the effects of different sources of albumin on population doubling times and colony forming ability; the latter is considered a measure of stemness in stromal cell cultures [5]. To further investigate the differences detected we compared levels of bound fatty acids (FAs) and the effect of the stabilizing compound octanoate. Finally we measured levels of the membrane localized Na^+^/K^+^ ATPase in cultured mesenchymal stromal cells, a previously identified target of octanoate [6].

## 2. Material and Methods

### 2.1. Bone Marrow Mesenchymal Stromal Cell Isolation

Bone marrow aspirates were obtained from three healthy female donors with full ethical consent and approval from the Central Health and Disability Ethics Committee of New Zealand, in accordance with the Declaration of Helsinki [7]. All donors were negative for Hepatitis B and Hepatitis C and HIV. Donor 1 was 23 years old; donor 2 was 22 years old; donor 3 was 26 years old. Marrow aspirates were taken from the posterior iliac crest using 11 or 16 ga needles, following administration of 1% lignocaine local anesthetic. Aspirates were then diluted into *α*-MEM media with 10% human AB serum (NZ Blood Service) and 10 u/mL heparin (Sigma). Mononuclear cells were isolated from the diluted aspirates using density gradient centrifugation as previously described [8]. The collected mononuclear cells were then grown in *α*-MEM media with 10% human AB serum without heparin through the initial passage 0, to enable adaption to* ex vivo* culture. Aliquots of these cells were then frozen in media with 10% DMSO (Sigma) as cryoprotectant and stored in liquid nitrogen until required.

### 2.2. Mesenchymal Stromal Cell Culture

To examine the effects of different albumin preparations on the stored MSCs, frozen cell aliquots were thawed into PPRF-msc6 media substituted with the albumin of interest at a concentration of 4 g/L [4]. PPRF-msc6 consisted of DMEM/F12, glutamine 4 mM, NaHCO_3_ 20.5 mM, lipid concentrate 0.1% (all from Gibco), HEPES 4.9 mM, bovine insulin 23 mg/L, human apotransferrin 25 mg/L, putrescine 56 *μ*M, progesterone 18 nM, bovine fetuin 1 g/L, hydrocortisone 100 nM, ascorbic acid 2-phosphate 200 *μ*M (all from Sigma), human albumin 4 g/L (InVitroCare or CSL Behring), recombinant human FGF 2 *μ*g/L, and recombinant human TGF-*β* 1 *μ*g/L (both from R&D Systems). Growth flasks were precoated for 30 minutes with 0.1% bovine gelatin (Sigma) when using PPRF-msc6 to promote surface adhesion. Cells were grown for two passages with a seeding density of 5000 cells/cm^2^ in PPRF-msc6 supplemented with the albumin of interest before experimental analyses were performed in an identical manner to that previously described [4]. DMEM supplemented with 10% fetal calf serum was used as reference media due to its widespread use in MSC culture. When using DMEM supplemented with 10% fetal bovine serum as growth media all conditions were the same with the exception that gelatin coating of culture flasks was not required. Harvest between passages was performed using TrypLE (Gibco) rather than trypsin-EDTA for all media.

### 2.3. Albumin

Three human albumin preparations were used: Cohn-fractionated albumin with octanoate and pasteurization (Cohn, InVitroCare), chromatographically purified albumin with octanoate and pasteurization (C-PO, CSL) and nonpasteurized, octanoate-free, chromatographically purified albumin (C-NPOF, CSL) obtained from the fractionation sequence prior to addition of octanoate. Albumin concentration in media was 4 g/L.

### 2.4. Immunophenotyping

To confirm MSC phenotype after growth in PPRF-msc6 or DMEM 10% FCS, cells were tested for the presence and absence of markers as recommended by the International Society for Cellular Therapy [9]. We used the BD Human MSC analysis kit (BD Biosciences), according to the manufacturers recommendations. In brief, MSCs were incubated with conjugated antibodies for the MSC markers, CD44-PE, CD166-PE, CD90-FITC, CD105-Per-CP, and CD73-APC. Markers absent from MSCs were screened using a cocktail of PE-conjugated CD45, CD34, CD116, CD19, and HLA-DR. Samples were analyzed on an Accuri C6 flow cytometer (BD Biosciences) and data processed using Accuri C6 software.

### 2.5. Differentiation

To verify that MSCs could undergo adipogenic or osteogenic differentiation, cells were induced to differentiate using specific differentiation media. Cells were plated into 24-well plates in triplicate at 2 × 10^4^ cells/cm^2^ and the following day differentiation media added. For adipogenic differentiation media were high glucose DMEM (Life Tech) with 10% FCS (Moregate NZ), insulin 4 *μ*g/mL, 3-isobutyl-1-methylxanthine 500 *μ*M, indomethacin 100 nM, and dexamethasone 1 *μ*M (all from Sigma), while osteogenic induction was DMEM with 1 g/L glucose (Life Tech), 10% FCS, *β*-glycerophosphate 10 mM, dexamethasone 100 nM, and ascorbic acid 200 *μ*M (all from Sigma). Media were changed every 3 days and cells were fixed with 4% paraformaldehyde after 18 days (adipogenesis) or 21 days (osteogenesis). Lipid droplets (adipogenesis) were stained with oil red-O [10] and calcium containing matrix (osteogenesis) stained with alizarin red [11].

### 2.6. Population Doubling Times

To determine population doubling times of cells grown in PPRF-msc6 containing different albumins or in DMEM 10% FCS, cells were plated into triplicate 96-well plates at 5000 cells/cm^2^ in respective media. Plates were fixed for 20 minutes with 4% paraformaldehyde at 24 and 72 hours. Cell numbers were calculated by nuclear staining with Hoechst 33342 and automated counting of micrographs using Image-J [12]. Population doubling times were calculated from the 24- and 72-hour time points using the following formula: *dt* = *t* × ln⁡2/ln⁡(*C*_*t*_/*C*_0_), where *dt* is the doubling time, *t* is the time between cell counts, *C*_0_ is the 24-hour cell count, and *C*_*t*_ is the 72-hour cell count [13].

### 2.7. Colony Forming Ability

Cells grown in either DMEM 10% FCS or PPRF-msc6 with different albumins were used to measure colony forming ability. Cells were harvested and counted using a Scepter hand-held cell counter (Millipore) and a total of 100 cells plated in triplicate into *α*-MEM (Life Tech), 10% fetal calf serum (Moregate Serum, New Zealand) medium in a p100 culture dish (Falcon). 14 days later cells were fixed with methanol and stained using 0.1% crystal violet and colonies counted.

### 2.8. Albumin Bound Fatty Acid Characterization

To measure the amount and type of fatty acids in the albumin preparations 1.5 g samples were freeze-dried and spiked with methyl tricosanoate (23:0, 0.03 mg) as an internal standard, and fatty acids converted to fatty acid methyl esters (FAME) by direct transmethylation [14]. 1% sodium methoxide (1.5 mL) was added and the sample heated in a Teflon-lined screw cap test tube at 80°C for 20 min. After cooling, 5% HCl in methanol (3 mL) was added and the sample heated at 80°C for 20 min. After cooling, water (0.75 mL) was added and FAME were extracted into hexane (2 mL). Gas chromatography was performed using a TG-WAX MS column 30 m length × 0.25 mm id × 0.25 *μ*m film thickness at an oven temperature of 200°C, at a constant column flow of 2.7 mL/min. Detection was by flame ionization. Fatty acid identification was by comparison to standards and by using equivalent chain lengths [15].

### 2.9. Octanoate Quantitation

To measure the amount of octanoate in albumin samples, 1 mL aliquots were spiked with 2-ethylhexanoic acid (1 mg) as an internal standard and extracted with 5 mL of n-heptane and 1 mL of 20% citric acid. Gas chromatography was performed using a megapore column 30 m length × 0.53 mm id × 1 *μ*m film thickness at an oven temperature of 180°C and a constant column flow of 3.7 mL/min. Detection was by flame ionization.

### 2.10. Immunofluorescence Labelling of Na^+^/K^+^ ATPase

Cells were grown from passage 1 through to passage 4 as described earlier. Two growth media were used, PPRF-msc6 with NPOF and the same media with 0.64 mM added octanoate. Passage 4 cells were grown on coverslips coated with gelatin until about 50% confluent. Coverslips were then fixed with 4% paraformaldehyde for 15 minutes. Antigen retrieval was performed for 30 minutes at 100°C in 10 mM Tris, 1 mM EDTA, pH 9.0. Blocking was carried out with PBS containing 10% normal goat serum, 0.1% bovine serum albumin, 0.3 M glycine, and 0.1% Tween-20. The Na^+^/K^+^ ATPase antibody, *α*6F (DSHB, Iowa University), was incubated with cells overnight at a dilution of 1 : 50 in blocking buffer. Visualization of *α*6F was performed with goat anti-mouse-Alexa 488 (Invitrogen) diluted at 1 : 250. Slides were viewed on an Olympus BX51 fluorescent microscope.

### 2.11. Western Blotting

To measure levels of Na^+^/K^+^ ATPase, cells grown for 4 passages in media containing C-NPOF albumin with or without 0.64 mM octanoate were lysed in RIPA buffer (Sigma) and protein was quantitated by the bicinchoninic acid method. 12 *μ*g of lysate was separated on a 10% SDS-polyacrylamide gel and subsequently transferred to PVDF membrane. Na^+^/K^+^ ATPase was detected with *α*6F at 1 : 100 dilution and visualized using goat-anti-mouse HRP and chemiluminescence. An antibody against *β*-actin (Sigma) was used to normalize densitometric analysis of lanes.

### 2.12. Statistical Analysis

Population doubling times were calculated from individual experiments and compared using Student's *t*-tests with Microsoft Excel. Standard error was calculated in a similar manner using respective *n* values and individual experiments were performed at least 5 times. Individual data from all donors were combined in these analyses to increase the variance and hence the power of the *t*-tests. Colony forming experiments were analyzed in a similar way except using raw colony counts with a minimum of 3 experiments per donor. Western blotting band densities were calculated using Image-J, corrected for loading using *β*-actin band densities and values tested for significance using Student's *t*-test.

## 3. Results & Discussion

### 3.1. Immunophenotype and Differentiation of MSCs

**Figure 1:**
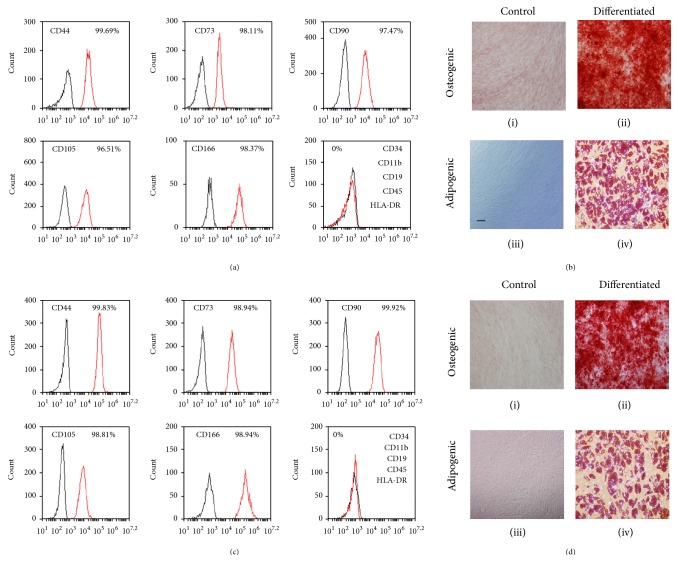
MSCs cultured in PPRF-msc6 with Cohn-fractionated albumin containing media (a) or DMEM 10% FCS (c) were analyzed for markers using flow cytometry. Cells grown in either medium were positive for CD44, 73, 90, 105, and 166 and negative for CD34, 11b, 19, 45, and HLA-DR. MSCs could differentiate into both osteoblasts and adipocytes when grown in either PPRF-msc6 with Cohn-fractionated albumin (b) or DMEM 10% FCS (d). Osteogenic calcium containing matrix laid down is stained with alizarin red (ii) while control (i) is negative. Lipid droplets in the adipocytes are stained with oil red-O and nuclei with Hoechst-33342 (iv) and are absent in the control (iii). Bar is equal to 100 *μ*m.

MSC populations grown from each of the 3 donors were characterized by flow cytometric immunophenotyping and in all media were found to be positive for CD 44, 73, 90, 105, and 166, (more than 95% positive cells) and negative (less than 5% positive cells) for CD45, 34, 116, 19, and HLA-DR (Figures 1(a) and 1(c)). Cells in all media could undergo adipogenic and osteogenic differentiation (Figures 1(b) and 1(d)), and no obvious differences in these capabilities were detected. Cells were also plastic adherent when grown in DMEM-10% fetal calf serum. Cells grown in PPRF-msc6 were also plastic adherent but gelatin coating of the flasks gave a more consistent result as previously shown by Jung et al. [4]. Taken together these results confirm that MSCs can be cultured in both DMEM 10% FCS and PPRF-msc6, regardless of the albumin preparation used.

### 3.2. Albumin Affects Population Doubling Time and Colony Forming Ability

**Figure 2:**
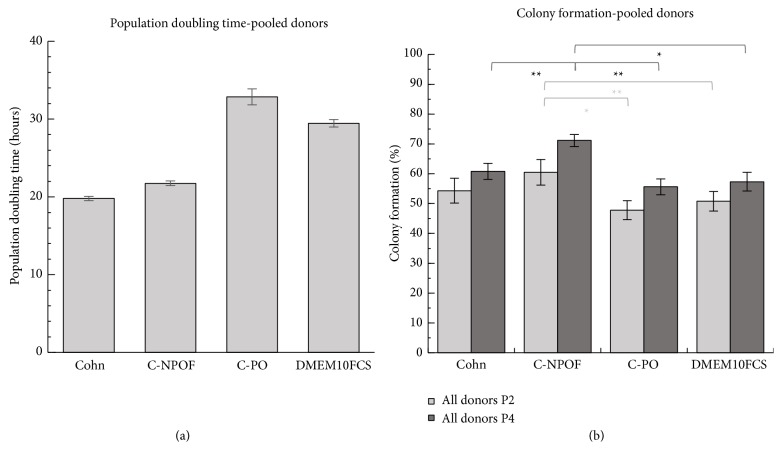
Growth and colony forming ability of donor MSCs. Pooled results represent the averages of three separate donors. Donor MSCs were grown in PPRF-msc6 with various albumins or in DMEM 10% FCS for two passages before growth analysis in a 96-well plate based assay. All population doubling times were significantly different to each other (*P* < 0.01) and were calculated from cell numbers at 24 and 72 hours (a). Colony forming ability was measured following similar growth conditions for either 2 or 4 consecutive passages (b). Results are combined from 3 separate donors and experiments repeated at least twice. Error bars represent standard error of the mean, ∗∗ indicates a *P* value of <0.01, and ∗ indicates a *P* value of <0.05.

Two properties that are fundamental to amplifying MSCs* in vitro* are population doubling times (growth rates) and colony forming ability which is often used as a proxy for the degree of “stemness.” We used a 96-well plate assay combined with nuclear counting to measure growth of cells and to calculate population doubling times. We identified differences in MSC growth rates across the 3 albumins in all donors examined, with a high level of significance (*P* < 0.01) when tested using Student's *t*-test (Figure 2(a)). PPRF-msc6 medium has a clear advantage in achieving higher growth rate when using Cohn-fractionated albumin or C-NPOF, when compared to the widely used DMEM supplemented with 10% fetal calf serum, or with PPRF-msc6 supplemented with C-PO.

A faster population doubling time is desirable when growing MSCs for clinical application but also important is their colony forming capacity, as this property is a measure of the stemness of the cell population. Maintenance of colony forming ability of MSCs is likely to be reflected in capacity to produce higher total numbers of cells for cell therapy applications. It is also likely to be an important issue in retaining functional status of MSCs as differentiation capacity is lost when cells approach senescence* ex vivo.* Thus, colony forming ability may be a more important characteristic of culture media than capacity to support rapid growth. Interestingly, the colony forming ability varied, with C-NPOF-containing medium outperforming C-PO medium and DMEM 10% FCS after two passages. When the analysis was repeated with additional two passages (i.e., 4 passages in respective media), C-NPOF albumin enhanced CFU ability above all others (*P* < 0.05). Previous work with MSC culture has demonstrated that the colony forming ability is separable from the growth rate. For example, platelet derived growth factor can enhance growth rate but it lowers colony forming ability [4]. These results suggest that PPRF-msc6 media supplemented with C-NPOF or with Cohn albumin result in an acceptable combination of growth rate and colony forming ability when compared to the remaining media.

### 3.3. Albumin Associated Fatty Acids

**Figure 3:**
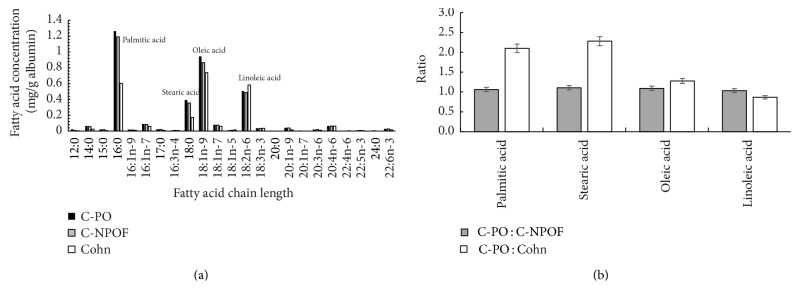
Lipid profiles of Cohn-fractionated albumin, C-PO, and C-NPOF. Levels of fatty acids associated with each albumin preparation per gram of albumin plotted against the estimated chain length following gas chromatography. Major peaks identified with common names (a). Ratios of significant fatty acids C-PO : C-NPOF and C-PO : Cohn demonstrate the similarity of C-PO and C-NPOF and the dissimilarity of C-PO and Cohn (b).

We wondered whether these differences between albumins were related to their associated cargoes and looked at fatty acid (FA) content using fatty acid methyl ester analysis and gas chromatography. The results shown in Figure 3 indicate an almost identical lipid profile between C-PO and C-NPOF (obtained from different batches of the same production process) indicating that the fatty acid profile is not associated with the functional differences observed. Interestingly the Cohn-fractionated albumin has a different FA profile to C-PO and C-NPOF and in general has less FAs (Figure 3(a)), but with a greater reduction in some shorter chain fatty acids. It would seem likely that the process of ethanol fractionation may extract albumin bound lipids to varying extents when compared to chromatographic methods. Comparison of the ratios of the individual FAs between albumins (Figure 3(b)) further suggests that the two purification methods result in changes to the relative amounts of individual FAs. The Cohn albumin has a different cargo of FAs and probably also other components such as metals, hormones, and growth factors when compared to C-NPOF. These differences may explain why the observed population doubling time is less than that of C-NPOF despite Cohn albumin possessing a low amount of octanoate (see later). It is interesting that palmitate derived from albumin is present between 2.4 and 4.8 mg/L in PPRF-msc6 which contributes significantly more of this lipid to the medium than that added in the lipid concentrate component. It should be noted also that palmitate has been shown to cause apoptosis in MSCs at concentrations of 125 *μ*M [16], which is about 5-fold higher than the concentration in PPRF-msc6 with C-PO, when quantitated in the final medium. The effects of these associated FAs on MSC health certainly warrant further research.

### 3.4. Effects of Octanoate on MSCs

**Figure 4:**
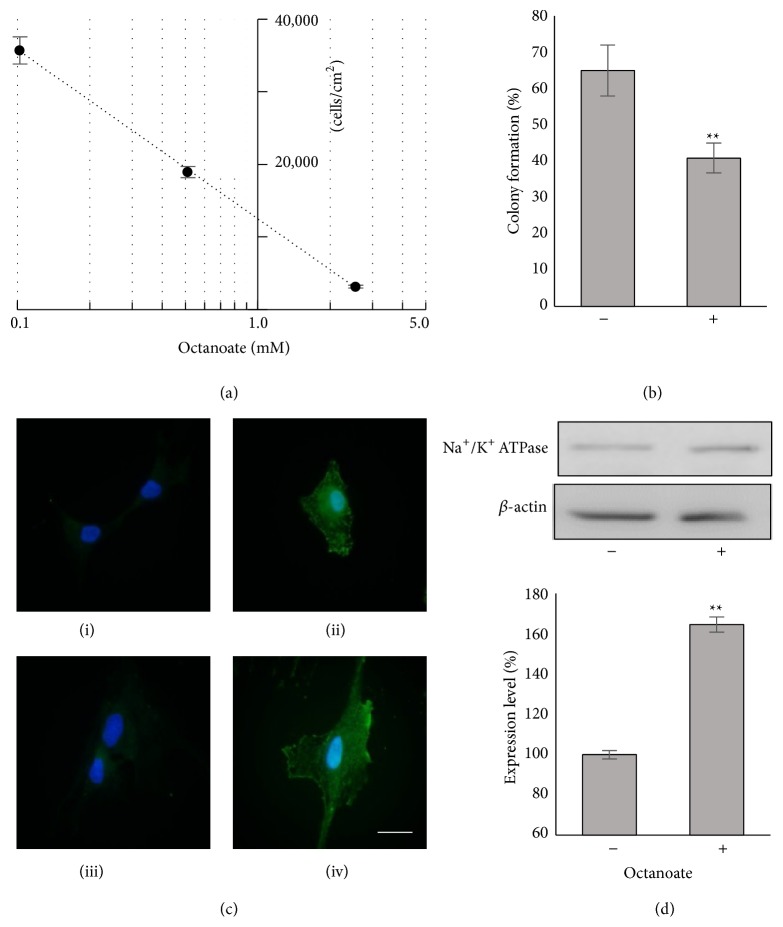
The effects of octanoate on MSCs. Growth of MSCs is inhibited at levels of octanoate in albumin products that are relevant to cell culture. Cells were grown for 3 days in PPRF-msc6 media with C-NPOF albumin and varying amounts of octanoate before counting. An inhibitory effect of 50% is seen at octanoate levels between 0.5 and 0.6 mM (a). Colony formation is reduced to about 40% of cells grown in PPRF-msc6 with C-NPOF albumin, 0.67 mM octanoate for 4 passages (b). MSCs grown for 4 passages in PPRF-msc6 with C-NPOF albumin ((i), (ii)) and C-NPOF albumin with octanoate ((iii), (iv)) express Na^+^/K^+^ ATPase. Panels (ii) and (iv) contain mouse anti-Na^+^/K^+^ ATPase 1 : 50 while all panels contain secondary goat anti-mouse antibody coupled to Alexa Fluor 488 (green). Nuclei are stained blue with Hoechst 33258. Bar equals 20 *μ*m (c). Quantitation of Na^+^/K^+^ ATPase expression in MSCs. Cells were grown for four passages as in (c) before being lysed in RIPA buffer and analyzed by Western blotting. Bands were quantitated using Image-J and values normalized against *β*-actin. Experiments were performed in duplicate (d). ∗∗ indicates a *P* < 0.01.

Given the near identity of the lipid profiles of C-PO and C-NPOF and the disparity between their growth and colony forming ability we hypothesized that the presence of octanoate in C-PO was detrimental to MSCs. Using Cohn albumin to further investigate the role of octanoate was precluded as we lacked an octanoate-free version of this albumin. However we could examine the growth inhibitory effects of octanoate on cells by comparing C-NPOF against C-NPOF with added octanoate. We tested growth inhibitory effects in a 96-well plate assay. Cells were grown for 3 days in the presence of various concentrations of octanoate before being counted (Figure 4(a)). Octanoate clearly inhibits MSC growth with a 50% inhibitory concentration between 0.5 and 0.6 mM. We then used gas chromatography to quantitate levels of octanoate in the albumins. C-PO contained 168.5 *μ*mol octanoate/g albumin, Cohn albumin had 83 *μ*mol/g, and C-NPOF had none. The concentration of octanoate in C-PO when diluted in medium is 0.67 mM. Based on this data the same amount of octanoate was added to C-NPOF as found in C-PO and cells grown to passage 4 in this medium. Colony formation was tested and found to be significantly reduced (Figure 4(b)). We demonstrated that both retention of higher levels of CFU and shorter doubling times over four culture passages are achieved in the absence of octanoate. Although we have shown that the addition of octanoate to human albumin preparations is detrimental to maintaining MSC characteristics, an alternative viral disinfection process that meets medicines licensing requirements for cell therapy products is now required [17]. Alternatively, a postpasteurization octanoate clean-up procedure followed by replacement of FA and other cargo molecules may be effective for albumin used in* ex vivo* MSC production.

### 3.5. Expression and Quantitation of Na^+^/K^+^ ATPase in MSCs

We were curious to identify potential targets of octanoate in MSCs and found that a previously identified membrane pump, Na^+^/K^+^ ATPase, was inhibited by octanoate in the cerebral cortex of young rats [6]. The Na^+^/K^+^ ATPase is a ubiquitous membrane localized pump that plays a major role in maintaining the internal ionic balance of cells. It works by using ATP hydrolysis to pump Na^+^ out and K^+^ into the cell, both against their concentration gradients. To investigate whether this potential target was also expressed in MSCs we used an antibody specific for the *α* subunit of the pump. We found the molecule was indeed expressed in MSCs with stronger staining localized to the plasma membrane and the nuclear envelope (Figure 4(c)), as expected. Further to this we grew MSCs for 4 passages in media containing C-NPOF albumin or C-NPOF albumin with added octanoate and quantitated Na^+^/K^+^ ATPase using *β*-actin as a loading control. Interestingly, we found an increased level of expression in the MSCs grown in the presence of octanoate. This observation could be explained by MSCs driving up the expression level of the pump in an attempt to compensate for the inhibition by octanoate. However, further work is required to measure functional capacity of Na^+^/K^+^ ATPase from cells grown in the presence and absence of octanoate. Relatively little is known about the relationship of the Na^+^/K^+^ ATPase to MSC stemness, but inhibitors of the pump have been shown to influence cell growth [18, 19] although the mechanisms remain unclear. The Na^+^/K^+^ ATPase is also involved in cellular differentiation [20] and is intricately linked to cellular signaling, cell volume, and intracellular pH [21] making it likely that this molecule contributes to MSC behavior* ex vivo*.

## 4. Conclusions

Human albumins derived from different preparation protocols result in significantly different growth and colony forming ability in human MSCs grown* ex vivo*. The presence of octanoate in human albumin preparations used in defined growth media for MSCs is not desirable. At concentrations found in commercially available albumin, octanoate reduces colony formation and increases population doubling time (slowing growth). Octanoate increases levels of Na^+^/K^+^ ATPase in human MSCs and it is possible this may lead to an alteration in stemness. We conclude that levels of octanoate in human albumin used for MSC culture should be reduced while acknowledging this will require innovative solutions to minimize infectious risk.

## Abbreviations

MSC: Mesenchymal stromal cell
C-NPOF: Chromatographically prepared, nonpasteurized, octanoate-free, human albumin
Cohn: Human albumin prepared by the Cohn fractionation process
PPRF-msc6: Pharmaceutical Production Research Facility-mesenchymal stromal cell media #6
hSA: Human serum albumin
FAs: Fatty acids
C-PO: Chromatographically prepared, pasteurized, octanoate added, human albumin
FAME: Fatty acid methyl esters
CFU: Colony forming unit.

## References

[1] Zaher W., Harkness L., Jafari A., Kassem M. (2014). An update of human mesenchymal stem cell biology and their clinical uses. *Archives of Toxicology*.

[2] (2014). *Albumin-Blood (Serum): MedlinePlus Medical Encyclopedia*.

[3] More J., Bulmer M. (2013). *Production of Plasma Proteins for Therapeutic Use*.

[4] Jung S., Sen A., Rosenberg L., Behie L. A. (2010). Identification of growth and attachment factors for the serum-free isolation and expansion of human mesenchymal stromal cells. *Cytotherapy*.

[5] Choumerianou D. M., Martimianaki G., Stiakaki E., Kalmanti L., Kalmanti M., Dimitriou H. (2010). Comparative study of stemness characteristics of mesenchymal cells from bone marrow of children and adults. *Cytotherapy*.

[6] De Assis D. R., Maria R. C., Ferreira G. C. (2006). Na^+^, K^+^ ATPase activity is markedly reduced by cis-4-decenoic acid in synaptic plasma membranes from cerebral cortex of rats. *Experimental Neurology*.

[7] World Medical Association (2013). World Medical Association declaration of Helsinki ethical principles for medical research involving human subjects. *JAMA*.

[8] Najar M., Rodrigues R. M., Buyl K. (2014). Proliferative and phenotypical characteristics of human adipose tissue-derived stem cells: comparison of Ficoll gradient centrifugation and red blood cell lysis buffer treatment purification methods. *Cytotherapy*.

[9] Dominici M., Le Blanc K., Mueller I. (2006). Minimal criteria for defining multipotent mesenchymal stromal cells. The International Society for Cellular Therapy position statement. *Cytotherapy*.

[10] Fink T., Zachar V. (2011). Adipogenic differentiation of human mesenchymal stem cells. *Methods in Molecular Biology*.

[11] Gregory C. A., Gunn W. G., Peister A., Prockop D. J. (2004). An Alizarin red-based assay of mineralization by adherent cells in culture: comparison with cetylpyridinium chloride extraction. *Analytical Biochemistry*.

[12] Rasband W. S. (2014). *Image J, US National Institutes of Health, Bethesda, Maryland, USA*.

[13] Yu S., Diao S., Wang J., Ding G., Yang D., Fan Z. (2014). Comparative analysis of proliferation and differentiation potentials of stem cells from inflamed pulp of deciduous teeth and stem cells from exfoliated deciduous teeth. *BioMed Research International*.

[14] Svetashev V. I., Vysotskii M. V., Ivanova E. P., Mikhailov V. V. (1995). Cellular fatty acids of Alteromonas species. *Systematic and Applied Microbiology*.

[15] Stránsky K., Jursík T., Vítek A. (1997). A Standard equivalent chain length values of monoenic and polyenic (methylene interrupted) fatty acids. *Journal of High Resolution Chromatography*.

[16] Lu J., Wang Q., Huang L. (2012). Palmitate causes endoplasmic reticulum stress and apoptosis in human mesenchymal stem cells: prevention by AMPK activator. *Endocrinology*.

[17] Che Y., Wilson F. J., Bertolini J., Schiff P., Maher D. W. (2006). Impact of manufacturing improvements on clinical safety of albumin: Australian pharmacovigilance data for 1988–2005. *Critical Care and Resuscitation*.

[18] Shank B. B., Smith N. E. (1976). Regulation of cellular growth by sodium pump activity. *Journal of Cellular Physiology*.

[19] Moreno Y Banuls L., Katz A., Miklos W. (2013). Hellebrin and its aglycone form hellebrigenin display similar in vitro growth inhibitory effects in cancer cells and binding profiles to the alpha subunits of the Na^+^/K^+^-ATPase. *Molecular Cancer*.

[20] Sayed M., Drummond C. A., Evans K. L. (2014). Effects of Na/K-ATPase and its ligands on bone marrow stromal cell differentiation. *Stem Cell Research*.

[21] Reinhard L., Tidow H., Clausen M. J., Nissen P. (2013). Na^+^,K^+^-ATPase as a docking station: protein-protein complexes of the Na^+^,K^+^-ATPase. *Cellular and Molecular Life Sciences*.

